# Factors Influencing Hormone Remission in Growth Hormone‐Secreting Pituitary Neuroendocrine Tumors With Residual Tumor: A Retrospective Cohort Study

**DOI:** 10.1111/cns.70574

**Published:** 2025-08-25

**Authors:** Yangyang Wang, Li Ma, Chuanbao Zhang, Shunchang Ma, Guijun Jia, Wang Jia, Xiudong Guan

**Affiliations:** ^1^ Department of Neurosurgery Beijing Tiantan Hospital, Capital Medical University Beijing China; ^2^ Beijing Neurosurgical Institute Capital Medical University Beijing China; ^3^ China National Clinical Research Center for Neurological Diseases (NCRC‐ND) Beijing China

**Keywords:** growth hormone, hormone remission, pituitary neuroendocrine tumors, surgery therapy, tumor residual

## Abstract

**Background:**

Growth hormone‐secreting pituitary neuroendocrine tumors (GH‐secreting PitNETs) pose significant health risks due to hormone‐related complications. Despite transsphenoidal surgical resection being the primary treatment, complete removal is often infeasible due to invasive growth patterns, leading to postoperative tumor residuals and uncertain hormone remission outcomes.

**Methods:**

This retrospective study included 458 patients with GH‐secreting PitNETs who underwent surgery at Beijing Tiantan Hospital. Data on preoperative hormone levels, MRI scans, and histopathological features were analyzed. Tumor segmentation, intratumor heterogeneity (ITH) scores, and subcluster clustering based on MRI data were computed using radiomic features, while multivariate analyses determined factors influencing hormone remission. Single‐cell data from four GH‐type pituitary adenomas were collected from public databases to explore ITH in GH1 gene expression.

**Results:**

Postoperative hormone remission was achieved in 61 of 144 patients (42.4%) with residual tumors. Univariate analysis demonstrated that in cases with tumor residuals, preoperative hormone levels, tumor resection rates, residual tumor volume, tumor residual location, residual‐tumor proximity to the internal carotid artery, and MRI‐based tumor heterogeneity were associated with hormone remission. Among these factors, preoperative hormone levels (10–30 ng/mL vs. ≤ 10 ng/mL: OR: 0.48, 95% CI 0.20–1.19, *p* = 0.115; > 30 ng/mL vs. ≤ 10 ng/mL: OR: 0.13, 95% CI: 0.04–0.36, *p* < 0.001), tumor resection rate (OR: 18.29, 95% CI: 2.08–160.97, *p* = 0.009), and tumor heterogeneity as measured by the ITH score (OR: 1.06, 95% CI: 1.00–1.12, *p* = 0.042) were independent predictors of hormone remission in cases with residual tumors. Moreover, single‐cell data showing highly variable GH1 expression within the same patient reveal ITH in hormone expression.

**Conclusion:**

Preoperative GH levels, tumor resection rates, and ITH scores independently predict hormone remission in GH‐secreting PitNETs with residuals. This will provide intraoperative decision‐making guidance on how to achieve the maximum possible hormone remission with residual tumors when complete tumor resection is not feasible.

## Graphical abstract

GH‐secreting pituitary adenomas exhibit spatial heterogeneity in hormone secretion, which may allow for hormone remission even in the presence of postoperative tumor residuals.

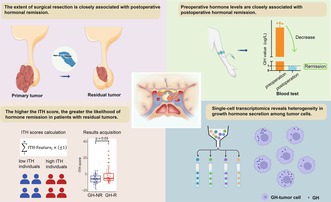

## 1 Introduction

Growth hormone‐secreting pituitary neuroendocrine tumors account for approximately 10% of pituitary neuroendocrine tumors (PitNETs), which rank among the most prevalent intracranial tumors in adults [1]. Growth hormone‐secreting PitNETs are linked to significant comorbidities and increased mortality risk [2]. Prolonged exposure to excessive growth hormone (GH) causes detrimental effects on various systems and organs, including the cardiovascular, respiratory, and musculoskeletal systems [3]. Moreover, patients with growth hormone‐secreting pituitary neuroendocrine tumors face a significantly higher risk of colorectal, breast, and thyroid cancers. As a result, their long‐term quality of life is generally poorer, with a lifespan reduced by approximately 30% compared to the general population [4, 5].

Transsphenoidal surgical (TSS) resection is recommended as the primary treatment for growth hormone‐secreting PitNETs [6, 7]. However, for large GH‐secreting macroadenomas, complete resection is often difficult, especially for tumors invading the bilateral cavernous sinuses, resulting in a postoperative hormone remission rate of only around 60% [8, 9, 10]. For patients whose hormone levels remain elevated after surgery, additional treatments such as medication, radiotherapy, or a second surgical procedure are required, often resulting in substantial financial strain [11, 12].

However, in many cases, tumor residuals are inevitable due to circumstances such as excessive intraoperative bleeding requiring termination of the surgery, or the tumor being too close to critical structures like the internal carotid artery or optic nerve [13, 14]. Forcing complete resection could lead to severe consequences, such as rupture of the internal carotid artery or optic nerve damage. In these difficult cases, we aim for maximal but safe resection to reduce postoperative hormone levels as much as possible, laying a solid foundation for subsequent endocrine and radiotherapy treatments [15].

Although tumor residuals are clearly a determining factor for hormone remission [16], previous studies have reported that 20%–28% of patients with residual tumors were still able to achieve hormone remission [17, 18, 19]. We also observed that a certain proportion of patients with residual tumors still achieved hormone remission in our collected cases. Investigating the factors that contribute to hormone remission in patients with residual tumors could aid in selecting the appropriate surgical approach and developing tumor resection strategies during surgery. This includes deciding where to resect, where to leave remnants, which areas to prioritize, and where to appropriately abandon resection, ensuring both successful surgery and postoperative hormone remission.

In this study, instead of treating tumor residual status as a single endpoint variable, we further refined it by examining residual location, residual volume, and extent of resection to explore factors influencing hormone remission in patients with residual tumors. Notably, a subset of patients with residual tumors still achieved hormone remission, suggesting spatial heterogeneity in hormone secretion—meaning that the residual tumor itself may not have high hormonal secretory activity. This hypothesis was supported by both MRI findings and single‐cell transcriptomic data. These insights may inform strategies to maximize postoperative hormone remission, particularly in cases where complete tumor resection is not feasible.

## 2 Methods

### 2.1 Data Collection

This retrospective study involved patients with GH‐secreting PitNETs, which was approved by the institutional review board of the Beijing Tiantan Hospital, affiliated with Capital Medical University. We retrospectively collected patients with PitNETs who underwent surgical treatment at Beijing Tiantan Hospital from 2020 to 2022. Inclusion criteria were as follows: (1) pathologically confirmed GH‐secreting PitNET; (2) serum GH level above the normal range; (3) IGF‐1 level exceeding the age‐ and sex‐adjusted upper normal limit. Exclusion criteria were as follows: (1) incomplete data, such as missing postoperative T1C‐enhanced imaging; (2) pituitary tumor apoplexy; (3) with prior intervention (surgical, pharmacological, or radiotherapeutic treatment).

Data on patient sex, age, and pathology information were carefully collected based on the medical record. The hormone collection process was conducted as follows: Preoperative GH and IGF‐1 levels were assessed through blood sampling around 8:00 a.m. after overnight fasting. Postoperative GH levels were measured through blood sampling at approximately 8:00 a.m. on the first postoperative day. Since GH levels measured 24 h after surgery strongly reflect long‐term hormone remission, follow‐up hormone assessments at 3 or 6 months were not continued [20]. Additionally, due to the long half‐life of IGF‐1, a downstream effector of GH, its level measured 24 h postoperatively cannot promptly reflect hormone remission [21]. According to our data, out of a total cohort of 458 patients, 367 had available IGF‐1 measurements within 24 h postsurgery. Among them, 116 patients did not achieve biochemical remission based on GH levels, while 251 did. In the 116 patients without GH remission, none had IGF‐1 levels that fell within the age‐ and sex‐adjusted normal range. Conversely, among the 251 patients who achieved GH remission, only 49 had normalized IGF‐1 levels based on age‐ and sex‐specific criteria. Therefore, the overall rate of discordance in hormone remission was as high as 55.04%, highlighting the limitation of early postoperative IGF‐1 as a remission marker. Therefore, postoperative IGF‐1 levels were not considered in the evaluation of hormone remission.

MRI data collection was performed twice, including preoperative and postoperative scans. The preoperative MRI was typically conducted within 2 months before surgery, while the postoperative MRI was performed approximately 3 days after surgery once the patient's condition stabilized. The evaluation of PitNET invasion was conducted along two dimensions. Tumors identified as Hardy's modified classification grades III or IV, or stages C, D, and E, as well as those categorized as Knosp classification grades III and IV, were defined as invasive. The determination of postoperative tumor residuals, including its location and its relationship with the internal carotid artery, was independently assessed by Yangyang Wang and Xiudong Guan through preoperative and postoperative imaging reviews. In cases of disagreement, senior neuroradiologist Wang Jia provided the final decision.

Previous studies have defined hormone remission in patients with acromegaly as follows: a random GH < 1 ng/mL or GH nadir < 0.4 ng/mL after oral glucose tolerance test (OGTT), with age‐ and sex‐adjusted normalization of IGF‐1 [22]. In this study, hormone remission was defined as a postoperative GH level below the upper limit of the normal reference range established by our medical center. The necessity and potential risks of using this definition are elaborated in detail in the discussion section.

### 2.2 MRI Data Processing and Tumor Segmentation

In this study, contrast‐enhanced T1‐weighted (CE‐T1) images were used for analysis. Tumor segmentation was conducted on axial CE‐T1 MR images for each patient using the ITK‐SNAP program (University of Pennsylvania, www.itksnap.org). One neuroradiologist delineated the three‐dimensional region of interest (ROI) of tumors manually. Another neuroradiologist carefully reviewed and verified the ROIs.

### 2.3 Heterogeneity‐Related Radiomic Features Extraction and ITH Scores Calculation

Radiomic features most closely associated with imaging intratumor heterogeneity (ITH) were selected as heterogeneity‐related features, based on the definitions provided by the PyRadiomics package (https://pyradiomics.readthedocs.io/en/latest/features.html). For first‐order features, those representing variations in intensity values were chosen, while for textural features, those reflecting heterogeneous or homogeneous texture patterns were selected. Ultimately, 42 radiomic features were identified as relevant to heterogeneity, including nine first‐order features and 33 texture features. Detailed definitions of these heterogeneity‐related features are provided in Table S1. The ITH score was subsequently calculated as the arithmetic sum of z‐score normalized features positively correlated with tumor heterogeneity, while the negatively correlated features were included as their inverse values [23]. We calculated the ITH score of each sample.

### 2.4 Local Features Extraction and Subcluster K‐Means Analysis

Local features, such as local entropy and energy values, along with 42 other features (detailed in Table S1), were obtained by analyzing each voxel within the ROI. A 3 × 3 × 3 moving window was employed to compute local features for each voxel, extracting 42 feature vectors per voxel. To segment the ROI into distinct subregions, the K‐means algorithm among the population was applied, resulting in the division of each sample into some unique subclusters. The number of subclusters was explored in a range of 3 to 8 to identify unique subclusters within the ROI. The Calinski‐Harabasz score was used to assess the clustering performance and determine the optimal cluster count. The results of five subclusters clustering were chosen for further analysis.

### 2.5 Single Cell Sequencing Data Collection and Processing

Single‐cell sequencing (scRNA‐seq) data from four GH‐positive PitNET cases were collected from public datasets [24]. The Seurat R package was utilized to analyze scRNA‐seq data [25]. Quality control was performed by filtering out low‐quality genes expressed in fewer than three cells, low‐quality cells with fewer than 100 identified genes, or cells with over 10% mitochondrial gene content. The remaining dataset was then normalized using the SCTransform method, effectively addressing batch effects. To reduce the dimensionality of the scRNA‐seq data, principal component analysis (PCA) was applied [26]. Specifically, 30 principal components were selected for further analysis using T‐distributed stochastic neighbor embedding (tSNE). As a result of these steps, a normalized gene expression matrix for each cell was generated.

### 2.6 Statistical Analysis

Normality of data distribution was assessed using the Shapiro–Wilk test before statistical analysis. The nonparametric Mann–Whitney U test was used for non‐normally distributed data, while the t‐test was applied to normally distributed data. Group comparisons of categorical variables were performed using Chi‐square and Fisher's tests. Multivariate analysis was performed using logistic regression analysis with the Enter method. The mediation package of R software was applied to conduct the regression‐based mediation analysis. All analyses were performed using R software version 4.0.1. All statistical tests were two‐tailed, and nominal P values below 0.05 were deemed statistically significant.

## 3 Results

### 3.1 Patient Characteristics

Table 1 presents the clinical and pathological characteristics of the study cohort. The mean age of the patients was 41.15 ± 11.22 years. There were 234 male and 224 female patients. Surgical approaches included 178 cases of microscopic transsphenoidal surgery (MTS), 256 cases of endoscopic transsphenoidal surgery (ETS), and 24 cases of transcranial surgery (TCS). Immunohistochemistry (IHC) hormone staining revealed 66 cases of GH‐only expression, 340 cases of single lineage (SL)‐multiple hormone expression, and 52 cases of multiple (ML)‐multiple hormone expression. Preoperative GH levels were distributed as follows: 126 cases with ≤ 10 ng/mL, 218 cases with 10–30 ng/mL, and 114 cases with > 30 ng/mL. The mean tumor volume was 8.06 ± 11.18 cm^3^, and residual tumors were observed in 144 cases, while 314 patients had no residual tumor. MRI revealed tumor invasion in 229 cases, while 229 cases showed no evidence of invasion.

**TABLE 1:** Characteristics of the 458 PitNETs.

Characteristic	Value
Age (year old)	
Mean ± SD	41.15 ± 11.22
Gender	
Male	234 (51.09%)
Female	224 (48.91%)
Surgery approach	
MTS	178 (38.86%)
ETS	256 (55.90%)
TCS	24 (5.24%)
IHC hormone	
GH‐only	66 (14.41%)
SL‐multiple hormone	340 (74.24%)
ML‐multiple hormone	52 (11.35%)
Preoperative GH level	
≤ 10 ng/ml	126 (27.51%)
10–30 ng/ml	218 (47.60%)
> 30 ng/ml	114 (24.89%)
Tumor volume (cm^3^)	
Mean ± SD	8.06 ± 11.18
Tumor residual	
Yes/No	144/314 (31.44/68.56%)
MRI invasion	
Yes/No	229/229 (50.00/50.00%)

Abbreviations: ETS, endoscopic transsphenoidal surgery; ML, multiple lineage; MTS, microscopic transsphenoidal surgery; SL, single lineage; TCS, transcranial surgery.

### 3.2 The Impact of Preoperative and Surgery‐Related Factors on Hormone Remission Among All Patients

We further analyzed potential factors that may influence hormone remission in clinical practice, including surgical approach, preoperative hormone levels, pathological type, and five other variables, totaling seven factors. We found that preoperative hormone levels, tumor invasion, the presence of residual tumors, and postoperative hormone remission were closely related (all *p* < 0.001) (Table 2). These findings are consistent with previous reports in the literature [27, 28]. Interestingly, 61 of 144 patients with residual tumors still achieved remission. Therefore, we proceeded to analyze potential factors influencing hormone remission in patients with residual tumors after PitNET surgery. These factors included preoperative hormone levels, the location of the residual tumor, its relationship with the internal carotid artery, tumor resection rate, residual tumor volume, and MRI heterogeneity of the tumor.

**TABLE 2:** Comparison of features between GH remission and nonremission groups among 458 PitNETs.

Characteristic	GH nonremission (*n* = 145)	GH remission (*n* = 313)	*p*
Surgery approach			
MTS	57 (39.31%)	121 (38.66%)	0.771
ETS	82 (56.55%)	174 (55.59%)	
TCS	6 (4.14%)	18 (5.75%)	
IHC hormone			
GH‐only	24 (16.55%)	42 (13.42%)	0.377
SL‐multiple hormone	109 (75.17%)	231 (78.80%)	
ML‐multiple hormone	12 (8.28%)	40 (12.78%)	
Preoperative GH level			
≤ 10 ng/ml	19 (13.10%)	107 (34.18%)	< 0.001
10–30 ng/ml	61 (42.07%)	157 (50.16%)	
> 30 ng/ml	65 (44.83%)	49 (15.66%)	
Tumor volume (cm^3^)			
Mean ± SD	9.20 ± 8.73	7.53 ± 12.12	0.137
Tumor residual			
Yes/No	83/62 (57.24/42.76%)	61/252 (19.49/80.51%)	< 0.001
MRI invasion			
Yes/No	101/44 (69.66/30.34%)	128/185 (40.89/59.11%)	< 0.001

Abbreviations: ETS, endoscopic transsphenoidal surgery; ML, multiple lineage; SL, single lineage; TCS, transcranial surgery; TS, microscopic transsphenoidal surgery.

### 3.3 The Impact of Preoperative and Surgery‐Related Factors on Hormone Remission Among Tumor Residuals

**FIGURE 1:**
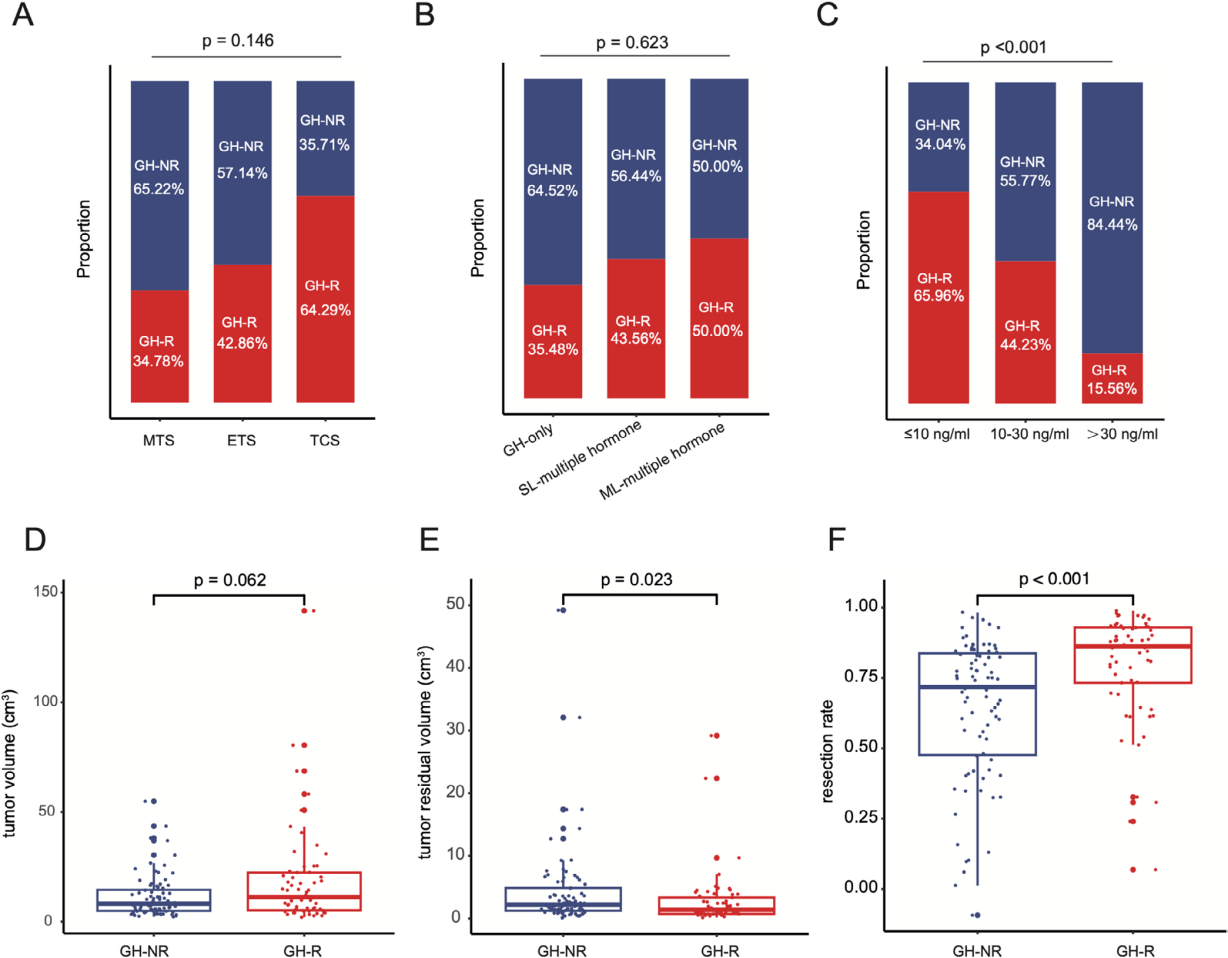
The relationship between hormone remission with residuals and operative approach (A), IHC group (B), preoperative serum GH level (C), tumor volume (D), tumor residual volume (E), resection rate (F). GH‐NR: Growth hormone nonremission; GH‐R: Growth hormone remission. MTS: Microscopic transsphenoidal surgery; ETS: Endoscopic transsphenoidal surgery; TCS: Transcranial surgery. SL: Single lineage; ML: Multiple lineage.

Univariate analysis revealed that among the 144 patients with residual tumors, the surgical approach, IHC hormone staining classification, and preoperative tumor volume were not significantly associated with postoperative hormone remission. However, there was a significant correlation with preoperative hormone levels, residual tumor volume, and tumor resection rate (Figure 1, Table 3). Higher preoperative hormone levels, larger residual tumor volumes, and lower resection rates were associated with worse hormone remission outcomes in patients with residual PitNETs.

**TABLE 3:** Comparison of features between GH remission and nonremission groups among PitNETs with residual tumors.

Characteristic	GH nonremission (*n* = 83)	GH remission (*n* = 61)	*p*
Surgery approach			0.146
MTS	30 (36.15%)	16 (26.23%)	
ETS	48 (57.83%)	36 (59.02%)	
TCS	5 (6.02%)	9 (14.75%)	
IHC hormone			0.623
GH‐only	20 (24.10%)	11 (18.03%)	
SL‐multiple hormone	57 (68.67%)	44 (72.13%)	
ML‐multiple hormone	6 (7.23%)	6 (9.84%)	
Preoperative GH level			< 0.001
≤ 10 ng/ml	16 (19.28%)	31 (50.82%)	
10–30 ng/ml	29 (34.94%)	23 (37.70%)	
> 30 ng/ml	38 (45.78%)	7 (11.48%)	
Tumor volume (cm^3^)			0.062
Mean ± SD	11.35 ± 9.75	18.70 ± 22.85	
Tumor residual volume (cm^3^)			0.010
Mean ± SD	4.34 ± 6.88	2.87 ± 4.67	
Tumor resection rate			< 0.001
Mean ± SD	64.1% ± 25.3%	79.3% ± 20.4%	
Suprasellar residual			0.459
Yes/No	29/54 (34.94/65.06%)	25/36 (40.98/59.02%)	
Intrasellar residual			0.044
Yes/No	15/68 (18.07/81.93%)	4/57 (6.56/93.44%)	
Parasellar residual			0.144
Yes/No	76/7 (91.57/8.43%)	51/10 (83.61/16.39%)	
Degree of ICA encasement			0.130
0	6 (7.23%)	9 (14.75%)	
0%–30%	31 (37.35%)	28 (45.90%)	
30%–50%	24 (28.92%)	16 (26.23%)	
> 50%	22 (26.50%)	8 (13.12%)	
MRI invasion			0.938
Yes/No	69/14 (83.13/16.87%)	51/10 (83.61/16.39%)	
ITH score	−4.27 ± 6.86	−1.04 ± 9.72	0.009
ITH subclusters	3.82 ± 0.73	4.11 ± 0.80	0.023

Abbreviations: ETS, endoscopic transsphenoidal surgery; ITH, intratumor heterogeneity; ML, multiple lineages; MTS, microscopic transsphenoidal surgery; SL, single lineage; TCS, transcranial surgery.

### 3.4 The Relationship Between Residual Location and Hormone Remission Among Tumor Residuals

The location of residual tumors was also associated with hormone remission. Tumor remnants in the suprasellar and parasellar regions did not significantly affect hormone remission, whereas intrasellar residual tumors frequently led to hormonal nonremission. We additionally found intrasellar residual tumors were closely associated with the resection rate (Figure S1A). Mediation analysis revealed a significant indirect effect of intrasellar residuals on hormone remission through the resection rate, while the direct effect was not statistically significant (Figure S1B). These findings suggest that the impact of intrasellar residuals on hormone remission is likely mediated by their influence on surgical resection rates.

**FIGURE 2:**
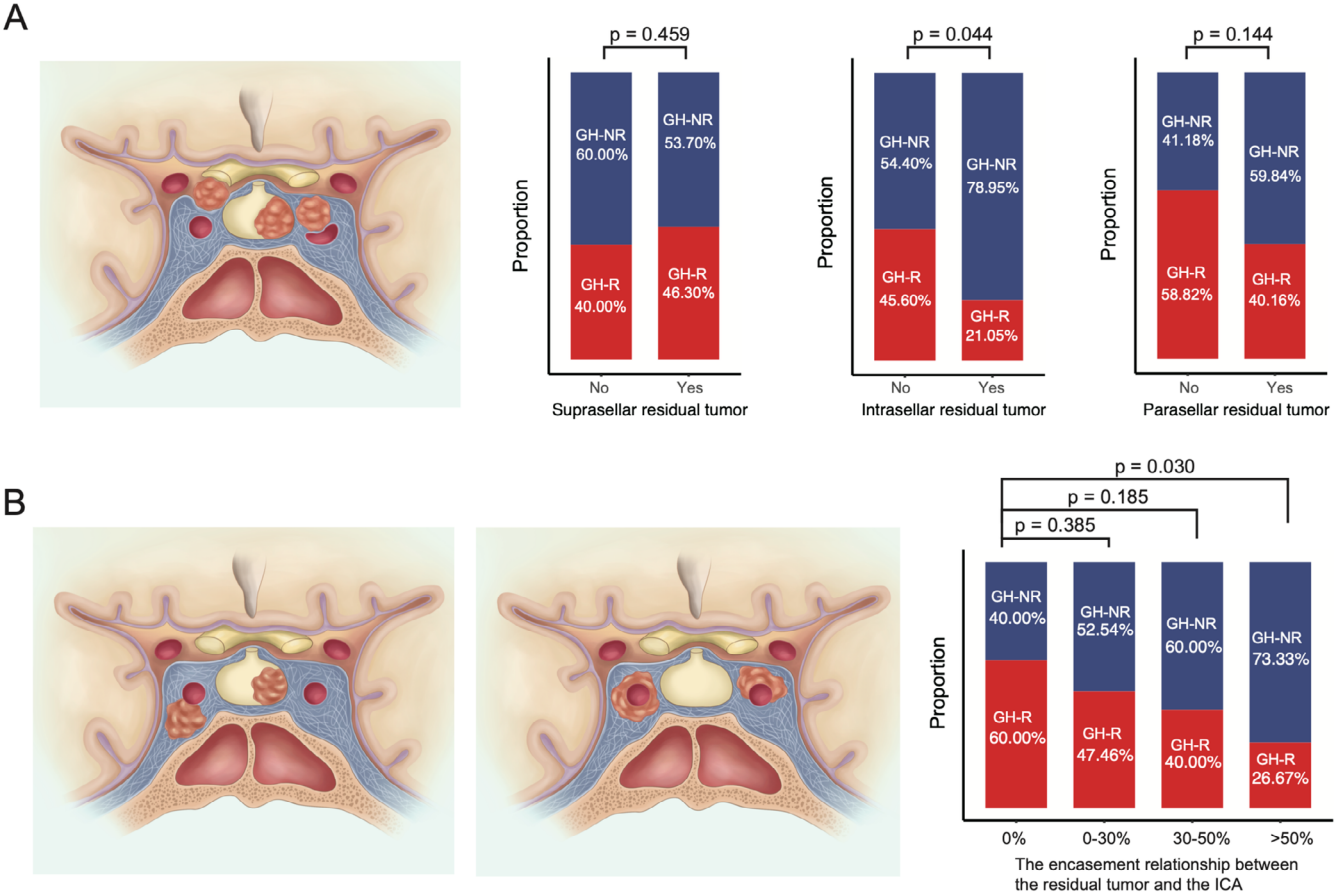
The relationship between hormone remission with residuals and residual location (A), degree of ICA encasement (B). GH‐NR: Growth hormone nonremission; GH‐R: Growth hormone remission. ICA: Internal carotid artery.

In addition to investigating the relationship between tumor location and hormone remission, we further explored the impact of tumor encasement of the internal carotid artery (ICA) on hormone remission (Figure 2A). We classified the tumor‐ICA relationship into four categories: (1) no apparent adjacency between the residual tumor and bilateral ICAs; (2) encasement of the ICA by the residual tumor with a combined encasement of less than 30% on both sides; (3) encasement of the ICA by the residual tumor with a combined encasement between 30% and 50% on both sides; (4) encasement of the ICA by the residual tumor with a combined encasement greater than 50% on both sides. We found that as the percentage of ICA encasement increased, the hormone remission rate of the residual tumor decreased. This indicates that the closer the residual tumor is to the ICA, the less likely hormone remission is to occur (Figure 2B).

### 3.5 The Impact of Tumor Heterogeneity on Hormone Remission Among Tumor Residuals

**FIGURE 3:**
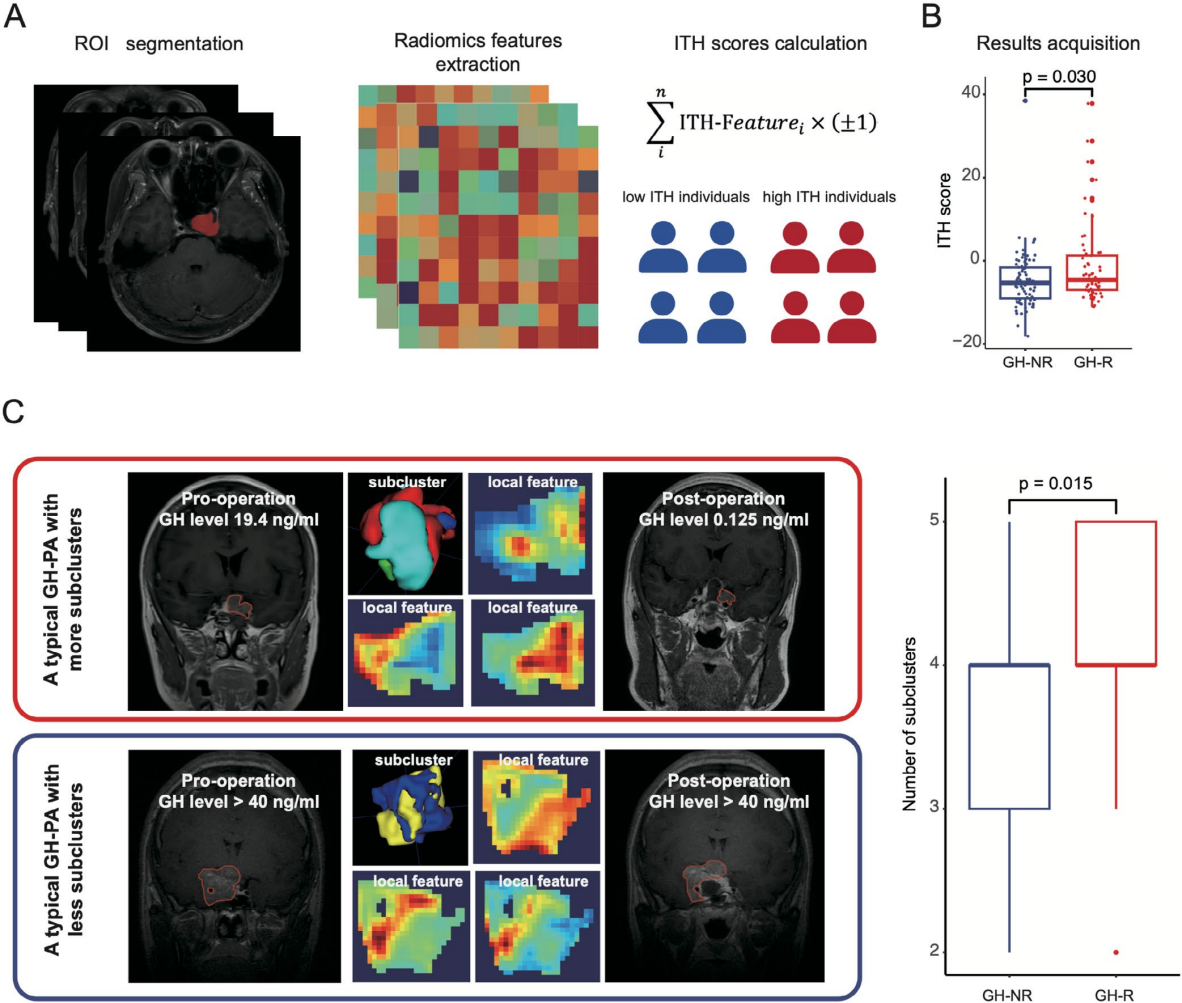
The relationship between hormone remission with residuals and intratumor heterogeneity. The flowchart of ITH scores calculation (A). Boxplots depicting the distribution of ITH scores between GH‐NR and GH‐R (B). Difference of subclusters and local features between GH‐NR and GH‐R (C). ITH: Intratumor heterogeneity. GH‐NR: Growth hormone nonremission; GH‐R: Growth hormone remission. PA: Pituitary adenoma.

The spatial heterogeneity of hormone secretion is a potential factor influencing hormone remission in cases with residual tumors. We defined the ITH as the arithmetic sum of z score normalized features positively related to tumor heterogeneity and the opposite value of features negatively related to tumor heterogeneity. We calculated the ITH score of each sample (Figure 3A). The ITH score in the residual hormone remission group was significantly higher than that in the nonremission group (*p* = 0.030) (Figure 3B). Additionally, clustering local features obtained from MRI data into different subclusters serves as another method to reflect tumor heterogeneity. A higher number of subclusters in a patient indicates greater tumor heterogeneity. Through unsupervised tumor clustering, we found that the number of subclusters per patient in the residual hormone remission group was significantly higher than in the nonremission group (Figure 3C). This finding aligns with the ITH score results mentioned above, suggesting that greater tumor heterogeneity may facilitate hormone remission in residual tumors, an interesting observation that will be discussed further.

### 3.6 Multivariable Analysis

To account for potential confounding factors and verify their independence, we included the factors that showed significant differences in postoperative hormone remission of residual tumors in a multivariate analysis. We found that high preoperative hormone levels, tumor resection rate, and tumor heterogeneity (ITH score) were independent factors influencing whether hormone remission occurred in residual tumors. The multivariate analysis results are summarized in Table 4. Preoperative GH level was significantly associated with outcomes (*p* < 0.001). Compared to the reference group (≤ 10 ng/mL), patients with GH levels of 10–30 ng/mL had an odds ratio (OR) of 0.48 (*p* = 0.115, 95% CI: 0.20–1.19); while those with GH levels > 30 ng/mL demonstrated a significantly reduced OR of 0.13 (*p* < 0.001, 95% CI: 0.04–0.36). The tumor resection rate was also significantly associated with remission outcomes, with an OR of 18.29 (95% CI: 2.08–160.97) (*p* = 0.009). In contrast, tumor residual volume (OR = 1.00, 95% CI: 0.99–1.01, *p* = 0.721) and the residual location (OR = 0.50, 95% CI: 0.13–1.85, *p* = 0.299) were not significant predictors. Tumor heterogeneity, measured by the ITH score, was significant with an OR of 1.06 (95% CI: 1.00–1.12) (*p* = 0.042), suggesting a potential association with outcomes. However, the number of subclusters did not show statistical significance (OR = 1.73, 95% CI: 0.71–4.58, *p* = 0.267). These findings highlight that preoperative GH levels, tumor resection rate, and tumor heterogeneity play key roles in influencing hormone remission outcomes, while other variables like residual volume and location were not significant predictors.

**TABLE 4:** Multivariate analysis of factors associated with GH remission outcomes among PitNETs with residual tumors.

Characteristic	OR (95% CI)	*p*
Preoperative GH level		
≤ 10 ng/ml	Ref.	
10–30 ng/ml	0.48 (0.20–1.19)	0.115
> 30 ng/ml	0.13 (0.04–0.36)	< 0.001
Tumor residual volume	1.00 (0.99–1.01)	0.721
Tumor resection rate	18.29 (2.08–160.97)	0.009
Intrasellar residual	0.50 (0.13–1.85)	0.299
ITH score	1.06 (1.00–1.12)	0.042
ITH subclusters	1.73 (0.71–4.58)	0.267

Abbreviation: ITH, intratumor heterogeneity.

### 3.7 ScRNA‐Seq Analysis of PitNETs

After applying the same preprocessing and data quality control standards, we obtained a total of 860 cells from the four samples, with individual cell counts of 95, 100, 525, and 140, respectively (Figure S2A,B). Among the 860 cells analyzed, those with high POU1F1 (PIT‐1) gene expression were identified as GH‐secreting tumor cells, totaling 828 cells (96.28%) (Figure S2C,D). The number of tumor cells identified in each of the four samples was 95, 100, 494, and 139, respectively (Figure S2E–H). Scatter plot analysis revealed marked differences in GH1 gene expression levels among tumor cells within each sample, highlighting the ITH of hormone secretion across individual cells within the same tumor (Figure S2I).

## 4 Discussion

The internationally accepted criterion for hormone remission in acromegaly includes a random GH < 1 ng/mL or GH nadir < 0.4 ng/mL after OGTT, with age‐ and sex‐adjusted normalization of IGF‐1 [22]. However, in our retrospective cohort (*n* = 458), remission rates using this standard were low: 25.11% overall and only 4.17% in patients with residual tumors (*n* = 144), limiting statistical comparisons. Applying our medical center‐specific GH reference range raised remission rates to 68.34% and 42.36%, respectively, allowing balanced group comparisons.

Adjusting the definition of hormone remission introduces methodological concerns, as differing criteria may select heterogeneous patient subgroups, potentially altering the strength and direction of associations between clinical predictors and outcomes. Patients classified as nonremitted under stricter standards may be reclassified as remitted under lenient ones, possibly diluting or inflating predictor effects. However, in our 458‐patient cohort, correlations between remission (based on our center's definition) and established prognostic factors remained consistent with previous literature [18, 29, 30], such as preoperative GH levels, tumor invasiveness, and surgical resection degree, suggesting robust findings. Moreover, although defining hormone remission as a postoperative GH level below the upper limit of our center's normal range does not strictly meet the conventional criteria for remission, this definition holds clinical value. It reflects a substantial reduction in GH burden and can be interpreted as partial remission or a treatment response. This not only provides a more realistic assessment of surgical efficacy but also establishes a favorable baseline for subsequent gamma knife or pharmacological therapy, especially in cases with inevitable residual tumors. Thus, our definition offers both analytical feasibility and practical clinical relevance.

Previous studies have primarily focused on exploring factors influencing preoperative hormone remission. For example, elevated preoperative GH levels are a significant predictor of reduced hormone remission. Patients with lower GH levels prior to surgery tend to achieve better biochemical control postoperatively [27]. Another example is that cavernous sinus invasion and suprasellar extension are critical factors associated with a lower rate of hormone remission. Tumors with invasive features are less likely to be completely resected, leading to higher residual rates and diminished postoperative hormonal control [28]. Besides, tumor remnants have been identified as one of the critical adverse factors affecting postoperative hormone remission [31], which is consistent with our findings from 458 cases. However, few studies have further explored the factors influencing hormone remission in patients with residual tumors. Our study further explored the factors associated with hormone remission among patients with tumor residuals.

Innovatively, we proposed that the location of the residual tumor and its relationship with the internal carotid artery are associated with hormone remission. Among residual tumor locations, intrasellar remnants were associated with lower hormone remission rates compared to those without intrasellar involvement. Mediation analysis supported that the key factor influencing hormone remission is not the anatomical location of the intrasellar remnant itself, but rather the fact that patients with intrasellar residuals often have larger residual tumor volumes. Additionally, the greater the degree of encasement of the ICA by the tumor, the less likely hormone remission becomes. The degree of ICA encasement by residual tumors reflects tumor invasiveness. Jean‐Philippe Cottier's study demonstrated that when MRI shows tumor encasement exceeding 67%, 100% of tumors breach the lateral wall of the cavernous sinus; when encasement is less than 25%, 100% of tumors do not breach this wall [32]. Thus, the degree of encasement is directly correlated with tumor invasiveness. Multiple prior studies have shown significantly elevated expression of vascular endothelial growth factor (VEGF) in invasive pituitary adenomas [33, 34], which promotes angiogenesis and reconstructs tumor blood circulation by bypassing the portal venous system [35]. Consequently, higher degrees of residual tumor encasement are associated with richer tumor blood supply, facilitating the entry of GH secreted by tumor cells into the circulatory system. This elevates GH levels and thereby increases the difficulty of achieving hormone remission.

Tumor heterogeneity is often associated with higher malignancy and poorer prognosis [23]. Traditionally, PitNETs have been considered benign tumors with relatively low heterogeneity. However, hormonal secretion heterogeneity in PitNETs does exist. For example, folliculostellate cells exist within PitNETs and exhibit clear spatial heterogeneity in their distribution. These cells mediate hormonal secretion, contributing to the spatial heterogeneity of hormone secretion within the tumor [36, 37, 38, 39]. For another example, GH‐secreting PitNETs are classified into densely granulated and sparsely granulated types at the histopathological level. The sparsely granulated type leads to uneven hormone distribution, resulting in spatially heterogeneous hormone secretion [40]. Furthermore, we collected single‐cell data from four cases of GH‐secreting PitNETs from previous studies and found significant expression variability of the key growth hormone gene GH1 among individual cells, highlighting the heterogeneity of hormone secretion by tumor cells [24]. These findings indicate that PitNETs exhibit regional heterogeneity in hormone secretion.

The concept of regional secretion homogeneity refers to the uniform distribution of hormone‐secreting functionality across the spatial structure of the tumor. In other words, every part of the tumor has the same capacity for hormone secretion. In this scenario, if tumor remnants remain after surgery, the residual tumor will continue secreting hormones, making it difficult for postoperative hormone levels to return to normal. In contrast, high regional secretion heterogeneity indicates that the tumor's hormone‐secreting function varies significantly across different spatial regions. Specifically, some parts of the tumor may not secrete hormones at all, some may have weak secretion capacity, while others may secrete hormones intensively. If the residual tumor after surgery happens to consist of nonsecreting or weakly secreting regions, postoperative hormone levels may decrease and achieve remission. Thus, the higher the regional secretion heterogeneity, the greater the likelihood of postoperative hormone remission in cases with residual tumors. This mechanism explains the potential relationship between tumor heterogeneity and hormone remission.

This study has several limitations that should be considered: (1) Lack of biological mechanism exploration: The study focuses on clinical outcomes without further investigation into underlying biological mechanisms. Future research should incorporate molecular or genetic analyses to uncover potential pathophysiological processes. (2) Short‐term hormone remission assessment: Hormone remission was evaluated based solely on short‐term outcomes, lacking long‐term follow‐up data. (3) The ITH score provides a global assessment of hormone secretion heterogeneity across the entire tumor based on preoperative MRI, but it does not enable precise localization of high hormone‐secreting regions. Further development of deep learning algorithms and preoperative MRI‐based localization models is needed to identify these functional subregions more accurately.

## 5 Conclusion

The hormone remission rate following surgery for GH‐secreting PitNETs is approximately 60%. The primary reason for this relatively low rate is the inability to achieve complete tumor resection. Complete removal is particularly challenging in cases of invasive or large tumors. In such situations, ensuring that postoperative hormone levels remain as close to normal as possible becomes crucial. For tumors with moderate preoperative hormone levels and high tumor heterogeneity, achieving maximal tumor resection during surgery increases the likelihood of hormone remission, even if residual tumors remain. This approach can significantly reduce the risk of damage to critical structures caused by aggressive attempts at total resection. These findings provide valuable guidance for selecting preoperative surgical approaches and developing intraoperative resection strategies. In the future, these insights may improve GH hormone remission rates in patients with tumors that are difficult to fully resect, thereby enhancing the overall hormone remission rate of surgical treatment.

## Author Contributions

Conception and design: Yangyang Wang, Xiudong Guan, and Wang Jia. Acquisition of data: Yangyang Wang, Li Ma. Analysis and interpretation of data: Yangyang Wang. Drafting the article: Yangyang Wang. Critically revising the article: Wang Jia. Reviewed the submitted version of manuscript: Xiudong Guan. Approved the final version of the manuscript on behalf of all authors: Wang Jia. Statistical analysis: Yangyang Wang. Administrative/technical/material support: Chuanbao Zhang, Shunchang Ma, Guijun Jia. Study supervision: Wang Jia, Xiudong Guan.

## Consent

All authors read and approved this final manuscript.

## Conflicts of Interest

The authors declare no conflicts of interest.

## Supporting information


**Table S1:** Heterogeneity‐related radiomics features.
**Figure S1:** Association between intrasellar residual tumor, resection rate, and hormone remission.
**Figure S2:** Results of Single‐Cell Sequencing.

## Data Availability

The data that support the findings of this study are available on request from the corresponding author. The data are not publicly available due to privacy or ethical restrictions.

## References

[1] M. E. Molitch , “Diagnosis and Treatment of Pituitary Adenomas: A Review,” JAMA 317, no. 5 (2017): 516–524. 28170483 10.1001/jama.2016.19699

[2] M. Fleseriu , B. M. K. Biller , P. U. Freda , et al., “A Pituitary Society Update to Acromegaly Management Guidelines,” Pituitary 24, no. 1 (2021): 1–3. 33079318 10.1007/s11102-020-01091-7 PMC7864830

[3] A. Colao , L. F. S. Grasso , A. Giustina , et al., “Acromegaly,” Nature Reviews. Disease Primers 5, no. 1 (2019): 20. 10.1038/s41572-019-0071-6 30899019

[4] J. C. Wu , W. C. Huang , H. K. Chang , C. C. Ko , J. F. Lirng , and Y. C. Chen , “Natural History of Acromegaly: Incidences, re‐Operations, Cancers, and Mortality Rates in a National Cohort,” Neuroendocrinology 110, no. 11–12 (2020): 977–987. 31822015 10.1159/000505332

[5] M. Terzolo , G. Reimondo , P. Berchialla , et al., “Acromegaly Is Associated With Increased Cancer Risk: A Survey in Italy,” Endocrine‐Related Cancer 24, no. 9 (2017): 495–504. 28710115 10.1530/ERC-16-0553

[6] S. Melmed , A. Colao , A. Barkan , et al., “Guidelines for Acromegaly Management: An Update,” Journal of Clinical Endocrinology and Metabolism 94, no. 5 (2009): 1509–1517. 19208732 10.1210/jc.2008-2421

[7] L. Katznelson , E. R. Laws, Jr. , S. Melmed , et al., “Acromegaly: An Endocrine Society Clinical Practice Guideline,” Journal of Clinical Endocrinology and Metabolism 99, no. 11 (2014): 3933–3951. 25356808 10.1210/jc.2014-2700

[8] D. P. Bray , S. Mannam , R. S. Rindler , et al., “Surgery for Acromegaly: Indications and Goals,” Frontiers in Endocrinology 13 (2022): 924589. 35992136 10.3389/fendo.2022.924589 PMC9386525

[9] A. Giustina , P. Chanson , D. Kleinberg , et al., “Expert Consensus Document: A Consensus on the Medical Treatment of Acromegaly,” Nature Reviews. Endocrinology 10, no. 4 (2014): 243–248. 10.1038/nrendo.2014.21 24566817

[10] A. E. Yildirim , M. Sahinoglu , D. Divanlioglu , et al., “Endoscopic Endonasal Transsphenoidal Treatment for Acromegaly: 2010 Consensus Criteria for Remission and Predictors of Outcomes,” Türk Nöroşirürji Dergisi 24, no. 6 (2014): 906–912. 10.5137/1019-5149.JTN.11288-14.1 25448208

[11] M. Fleseriu , F. Langlois , D. S. T. Lim , E. V. Varlamov , and S. Melmed , “Acromegaly: Pathogenesis, Diagnosis, and Management,” Lancet Diabetes and Endocrinology 10, no. 11 (2022): 804–826. 36209758 10.1016/S2213-8587(22)00244-3

[12] S. Melmed , “Pituitary‐Tumor Endocrinopathies,” New England Journal of Medicine 382, no. 10 (2020): 937–950. 32130815 10.1056/NEJMra1810772

[13] X. Ma , Y. Zhang , Z. J. Yang , et al., “Internal Carotid Artery Injury During Endoscopic Transsphenoidal Pituitary Surgery: Risk Factors, Management,” Neurochirurgie 70, no. 1 (2024): 101515. 38052154 10.1016/j.neuchi.2023.101515

[14] Ş. Hanalioğlu , İ. Işıkay , and M. Berker , “Splitting of the Optic Nerve by a Pituitary Macroadenoma,” World Neurosurgery 89 (2016): 35. 10.1016/j.wneu.2016.01.035 26805676

[15] N. A. Tritos and K. K. Miller , “Diagnosis and Management of Pituitary Adenomas: A Review,” JAMA 329, no. 16 (2023): 1386–1398. 37097352 10.1001/jama.2023.5444

[16] S. Sarkar and A. G. Chacko , “Surgery for Acromegaly,” Neurology India 68 (2020): S44–S51. 32611892 10.4103/0028-3886.287664

[17] S. C. Shen , C. C. Shen , T. W. Pu , and W. Y. Cheng , “Long‐Term Effects of Intracapsular Debulking and Adjuvant Somatostatin Analogs for Growth Hormone‐Secreting Pituitary Macroadenoma: 10 Years of Experience in a Single Institute,” World Neurosurgery 126 (2019): e41–e47. 30716503 10.1016/j.wneu.2019.01.125

[18] T. Cardinal , C. Collet , M. Wedemeyer , et al., “Postoperative GH and Degree of Reduction in IGF‐1 Predicts Postoperative Hormonal Remission in Acromegaly,” Frontiers in Endocrinology 12 (2021): 743052. 34867787 10.3389/fendo.2021.743052 PMC8637049

[19] J. L. Yan , M. Y. Chen , Y. L. Chen , et al., “Surgical Outcome and Evaluation of Strategies in the Management of Growth Hormone‐Secreting Pituitary Adenomas After Initial Transsphenoidal Pituitary Adenectomy Failure,” Frontiers in Endocrinology 13 (2022): 756855. 35498411 10.3389/fendo.2022.756855 PMC9048041

[20] E. H. Kim , M. C. Oh , E. J. Lee , and S. H. Kim , “Predicting Long‐Term Remission by Measuring Immediate Postoperative Growth Hormone Levels and Oral Glucose Tolerance Test in Acromegaly,” Neurosurgery 70, no. 5 (2012): 1106–1113. 22067418 10.1227/NEU.0b013e31823f5c16

[21] A. Giustina , P. Chanson , M. D. Bronstein , et al., “A Consensus on Criteria for Cure of Acromegaly,” Journal of Clinical Endocrinology and Metabolism 95, no. 7 (2010): 3141–3148. 20410227 10.1210/jc.2009-2670

[22] S. Frara , F. Maffezzoni , G. Mazziotti , and A. Giustina , “The Modern Criteria for Medical Management of Acromegaly,” Progress in Molecular Biology and Translational Science 138 (2016): 63–83. 26940387 10.1016/bs.pmbts.2015.10.015

[23] G. H. Su , Y. Xiao , C. You , et al., “Radiogenomic‐Based Multiomic Analysis Reveals Imaging Intratumor Heterogeneity Phenotypes and Therapeutic Targets,” Science Advances 9, no. 40 (2023): eadf0837. 37801493 10.1126/sciadv.adf0837 PMC10558123

[24] Y. Cui , C. Li , Z. Jiang , et al., “Single‐Cell Transcriptome and Genome Analyses of Pituitary Neuroendocrine Tumors,” Neuro‐Oncology 23, no. 11 (2021): 1859–1871. 33908609 10.1093/neuonc/noab102 PMC8563320

[25] A. Butler , P. Hoffman , P. Smibert , E. Papalexi , and R. Satija , “Integrating Single‐Cell Transcriptomic Data Across Different Conditions, Technologies, and Species,” Nature Biotechnology 36, no. 5 (2018): 411–420. 10.1038/nbt.4096 PMC6700744 29608179

[26] S. Lall , D. Sinha , S. Bandyopadhyay , et al., “Structure‐Aware Principal Component Analysis for Single‐Cell RNA‐Seq Data,” Journal of Computational Biology 25, no. 12 (2018): 1365–1373. 10.1089/cmb.2018.0027 30133312

[27] S. Melmed , “Medical Progress: Acromegaly,” New England Journal of Medicine 355, no. 24 (2006): 2558–2573. 17167139 10.1056/NEJMra062453

[28] S. Larkin , R. Reddy , N. Karavitaki , S. Cudlip , J. Wass , and O. Ansorge , “Granulation Pattern, but Not GSP or GHR Mutation, Is Associated With Clinical Characteristics in Somatostatin‐Naive Patients With Somatotroph Adenomas,” European Journal of Endocrinology 168, no. 4 (2013): 491–499. 23288882 10.1530/EJE-12-0864

[29] P. De , D. A. Rees , N. Davies , et al., “Transsphenoidal Surgery for Acromegaly in Wales: Results Based on Stringent Criteria of Remission,” Journal of Clinical Endocrinology and Metabolism 88, no. 8 (2003): 3567–3572. 12915637 10.1210/jc.2002-021822

[30] H. Babu , A. Ortega , M. Nuno , et al., “Long‐Term Endocrine Outcomes Following Endoscopic Endonasal Transsphenoidal Surgery for Acromegaly and Associated Prognostic Factors,” Neurosurgery 81, no. 2 (2017): 357–366. 28368500 10.1093/neuros/nyx020

[31] H. G. Vuong and I. F. Dunn , “Clinical and Prognostic Significance of Granulation Patterns in Somatotroph Adenomas/Tumors of the Pituitary: A Meta‐Analysis,” Pituitary 26, no. 6 (2023): 653–659. 37735314 10.1007/s11102-023-01353-0

[32] J. P. Cottier , C. Destrieux , L. Brunereau , et al., “Cavernous Sinus Invasion by Pituitary Adenoma: MR Imaging,” Radiology 215, no. 2 (2000): 463–469. 10796926 10.1148/radiology.215.2.r00ap18463

[33] M. Yilmaz , E. Vural , K. Koc , and S. Ceylan , “Cavernous Sinus Invasion and Effect of Immunohistochemical Features on Remission in Growth Hormone Secreting Pituitary Adenomas,” Turkish Neurosurgery 25, no. 3 (2015): 380–388. 26037177 10.5137/1019-5149.JTN.9347-13.1

[34] H. E. Turner , Z. Nagy , K. C. Gatter , M. M. Esiri , A. L. Harris , and J. A. Wass , “Angiogenesis in Pituitary Adenomas ‐ Relationship to Endocrine Function, Treatment and Outcome,” Journal of Endocrinology 165, no. 2 (2000): 475–481. 10810311 10.1677/joe.0.1650475

[35] J. Schechter , P. Goldsmith , C. Wilson , et al., “Morphological Evidence for the Presence of Arteries in Human Prolactinomas,” Journal of Clinical Endocrinology and Metabolism 67, no. 4 (1988): 713–719. 3417848 10.1210/jcem-67-4-713

[36] D. Voit , W. Saeger , and D. K. Lüdecke , “Folliculo‐Stellate Cells in Pituitary Adenomas of Patients With Acromegaly,” Pathology, Research and Practice 195, no. 3 (1999): 143–147. 10220793 10.1016/S0344-0338(99)80026-0

[37] I. Vajtai , A. Kappeler , and R. Sahli , “Folliculo‐Stellate Cells of “True Dendritic” Type Are Involved in the Inflammatory Microenvironment of Tumor Immunosurveillance of Pituitary Adenomas,” Diagnostic Pathology 2 (2007): 20. 17597515 10.1186/1746-1596-2-20 PMC1910595

[38] L. Delfin , O. Mete , and S. L. Asa , “Follicular Cells in Pituitary Neuroendocrine Tumors,” Human Pathology 114 (2021): 1–8. 33991528 10.1016/j.humpath.2021.05.002

[39] M. D. Ilie , A. Vasiljevic , M. Chanal , et al., “Intratumoural Spatial Distribution of S100B + Folliculostellate Cells Is Associated With Proliferation and Expression of FSH and ERα in Gonadotroph Tumours,” Acta Neuropathologica Communications 10, no. 1 (2022): 18. 35139928 10.1186/s40478-022-01321-y PMC8827287

[40] S. L. Asa , O. Mete , A. Perry , and R. Y. Osamura , “Overview of the 2022 WHO Classification of Pituitary Tumors,” Endocrine Pathology 33, no. 1 (2022): 6–26. 35291028 10.1007/s12022-022-09703-7

