# Supplementary material for: Factors Influencing Hormone Remission in Growth Hormone‐Secreting Pituitary Neuroendocrine Tumors With Residual Tumor: A Retrospective Cohort Study
Source: CNS Neurosci Ther. 2025 Aug 25;31(8):e70574. doi: 10.1111/cns.70574 (PMC12376067; doi:10.1111/cns.70574)
Supplement: Supplementary file 1 — Table S1: Heterogeneity‐related radiomics features. Figure S1: Association between intrasellar residual tumor, resection rate, and hormone remission. Figure S2: Results of Single‐Cell Sequencing. [file CNS-31-e70574-s001.pdf]

Supplementary Information

Factors Influencing Hormone remission in Growth Hormone-Secreting Pituitary Neuroendocrine Tumors with Residual Tumor: A Retrospective Cohort Study

Table S1. Heterogeneity-related radiomics features

Category	Feature	Coefficient
Firstorder	Uniformity	-1
Firstorder	Entropy	1
Firstorder	InterquartileRange	1
Firstorder	Range	1
Firstorder	MeanAbsoluteDeviation	1
Firstorder	RobustMeanAbsoluteDeviation	1
Firstorder	StandardDeviation	1
Firstorder	Kurtosis	1
Firstorder	Variance	1
GLCM	Autocorrelation	-1
GLCM	Contrast	1
GLCM	JointEntropy	1
GLCM	DifferenceEntropy	1
GLCM	DifferenceVariance	1
GLCM	IMC1	-1
GLCM	IMC2	-1
GLCM	MCC	-1
GLCM	ID	-1
GLCM	IDN	-1
GLCM	IDM	-1
GLCM	IDMN	-1
GLSZM	GrayLevelNonUniformity	1
GLSZM	GrayLevelNonUniformityNormalized	1
GLSZM	SizeZoneNonUniformity	1
GLSZM	SizeZoneNonUniformityNormalized	1
GLSZM	ZoneEntropy	1
GLRLM	GrayLevelNonUniformity	1
GLRLM	GrayLevelNonUniformityNormalized	1
GLRLM	RunLengthNonUniformity	1
GLRLM	RunLengthNonUniformityNormalized	1
GLRLM	RunEntropy	1
GLDM	SmallDependenceEmphasis	-1
GLDM	LargeDependenceEmphasis	-1
GLDM	GrayLevelNonUniformity	1
GLDM	DependenceEntropy	1
GLDM	DependenceNonUniformity	1
GLDM	DependenceNonUniformityNormalized	1
NGTDM	Coarseness	1
NGTDM	Contrast	1
NGTDM	Busyness	1
NGTDM	Complexity	1
NGTDM	Strength	1

Figure S1

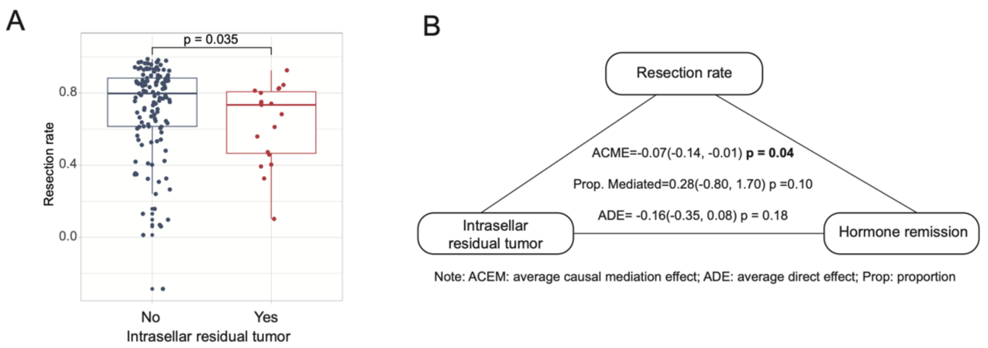

Figure S1. Association between intrasellar residual tumor, resection rate, and hormone remission. Patients with intrasellar residual tumors exhibited lower resection rates compared to those without **(A)**. Mediation analysis suggests that the effect of intrasellar residual tumors on hormone remission is primarily indirect, mediated through reduced resection rates, rather than a direct effect **(B)**.

Figure S2

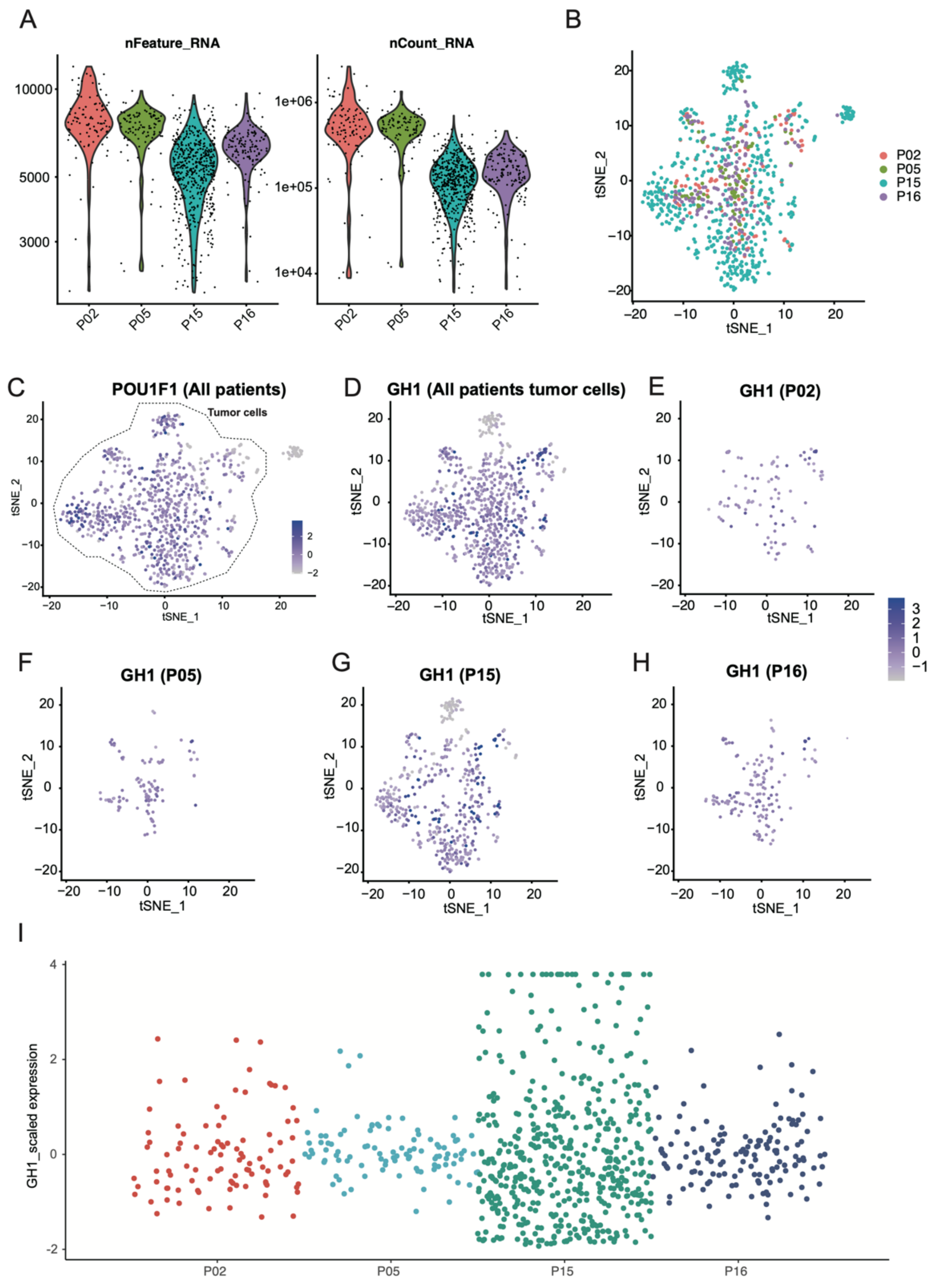

Figure S2. Results of Single-Cell Sequencing. The violin plots showing RNA features (nFeature_RNA) and RNA counts (nCount_RNA) across four patients **(A)**. A t-SNE plot of all 860 cells passed RNA QC from four patients **(B)**. Scatter plot showing a total of 828 tumor cells with highly POU1F1 expression **(C)**. Scatter plot showing highly variable GH1 expression within the same patient reveals intratumoral heterogeneity in hormone expression **(D-I)**.
